# Health-Related Physical Fitness and Arterial Stiffness in Childhood Cancer Survivors

**DOI:** 10.3389/fcvm.2019.00063

**Published:** 2019-05-15

**Authors:** Pia von Korn, Jan Müller, Christina Quell, Lisa Tenius, Renate Oberhoffer, Tobias Feuchtinger, Irene Schmid

**Affiliations:** ^1^Department of Prevention, Rehabilitation and Sports Medicine, University Hospital Klinikum Rechts der Isar, Technical University of Munich (TUM), Munich, Germany; ^2^Department of Preventive Pediatrics, Technical University of Munich, Munich, Germany; ^3^Dr. von Hauner University Children's Hospital, Pediatric Hematology and Oncology, Ludwig-Maximilians-University Munich, Munich, Germany

**Keywords:** health-related physical fitness, arterial stiffness, pulse wave velocity, childhood cancer survivors, cardiovascular health, prevention

## Abstract

**Introduction:** Despite decreasing mortality in pediatric oncology as a result of standardized treatment protocols, the high number of functional and cardiovascular late sequelae due to anticarcinogenic therapy remains unchanged. The aim of this study was to further assess functional limitations in Health-related Physical Fitness (HRPF) and cardiovascular risk by means of markers of arterial stiffness in Childhood Cancer Survivors (CCS).

**Materials and Methods:** Between March 2016 and August 2017 a total of 92 CCS (Age 12.5 ± 4.2 years, 43 girls) were recruited from their routine follow-up outpatient visit. HRPF was assessed using five Fitnessgram® tasks. Pulse Wave Velocity (PWV) along with peripheral and central blood pressure were assessed using oscillometric measurements performed by Mobil-O-Graph. Z-scores were used to compare the test results either to German reference values or to a recent healthy reference cohort.

**Results:** In CCS, the HRPF was significantly reduced (z-score: −0.28 ± 1.01, *p* = 0.011) as compared to healthy peers. The peripheral Systolic Blood Pressure (pSBP) was significantly increased (z-score: 0.31 ± 1.11, *p* = 0.017) and the peripheral Diastolic Blood Pressure (pDBP) was decreased (z-score: −0.30 ± 1.25, *p* = 0.040), resulting in an increased pulse pressure. The PWV (*p* = 0.649) and cSBP (*p* = 0.408), were neither increased nor showed any association to HRPF.

**Discussion:** CCS showed functional limitations in HRPF and an increased pulse pressure, which acts as an early onset parameter of arterial stiffness. Both a low HRPF and impaired hemodynamics are independent cardiovascular risk factors and needs to be taken into consideration in tertiary prevention of CCS.

## Introduction

Due to improved anticarcinogenic treatment regimens (chemotherapy and radiation) more children and adolescents are surviving a cancer diagnosis (1). As a result, there are a growing number of long-term adult survivors with multiple co-morbidities who need appropriate aftercare management. In numbers, more than 70% of the childhood cancer survivors (CCS) suffer from chronic health conditions (2), which are often directly associated to anticarcinogenic therapy. Improved screening modalities can predict cardiovascular morbidity in the future and are therefore essential.

Health related physical fitness (HRPF) is an indicator for the development, growths, and lifestyle of children and adolescents, and it is significantly reduced after acute treatment phase in CCS (3). Physical activity remains reduced in CCS for years after diagnosis (4), and frailty, a complex syndrome of the elderly, can occur prematurely in CCS (5).

Nevertheless, especially cardiovascular issues are also of particular concern in CCS as even low-dose anthracyclines, a group of chemotherapy drugs, can damage cardiomyocytes in the heart and impair endothelial functioning of the vessels (6, 7). The relative risk for coronary heart disease is therefore 10.4 times higher in CCS as compared to healthy siblings, and overall cardiovascular morbidity and mortality is seven-fold higher in CCS as compared to the normal population (8). Arterial stiffness, the ability of the vessels to expand and recoil, is a parameter for early cardiovascular aging and has been reported to be significantly higher in CCS than in the normal population (9, 10).

Previous studies outlined the beneficial association of HRPF and aerobic capacity to arterial vessel properties (11–13), but there is a lack of evidence-based data for CCS. This study therefore investigates functional outcomes in CCS by means of HRPF and arterial stiffness to determine lifelong cardiovascular risk factors.

## Patients and Methods

### Study Subjects

The study was conducted over two examination periods (March to June 2016 and February to August 2017) at the Department of Pediatric Hematology and Oncology in the Dr. von Hauner University Children‘s Hospital in Munich. Eligibility criteria were oncologic disease, current age of 6–20 years, absence of fever and acute infections, no mental retardation and the ability to write and communicate in German or English language without assistance. All participants could walk and do physical exercises without assistance. Physicians had to confirm the patients study participation. The study protocol (project number 724-16) was approved by the local Ludwig Maximilian University ethical board. Participation was voluntary and informed consent was obtained from the patients and parents after provision of oral and written information about the study procedures.

In total, 92 CCS (43 girls, age 12.5 ± 4.1 years) were examined for their HRPF and arterial stiffness.

The mean age at diagnosis was 8.8 ± 4.8 years and 54 of the patients were initially diagnosed with Leukemia, 28 with solid tumors and 10 with Lymphomas. This roughly correspond to the general prevalence of cancer diagnosis in children (1). Eighty-nine children were treated with chemotherapy, and 73 received anthracyclines with a mean dosage of 225 ± 83 mg/m^2^ body surface area. Ten children received radiotherapy where the mean radiation dosage was 28.4 ± 19.9 Gray (gy). Four Children were exposed to both chemotherapy including anthracyclines (248.0 ± 89.9 mg/m^2^), and radiation (18.4 ± 8.5 gy). Finally, three Children received neither chemotherapy nor radiotherapy (treated with surgery only or watch-and-wait).

All children were recruited during their regular post-acute treatment phase outpatient visit, 3.6 ± 2.8 years after primary cancer diagnosis.

### Anamnestic and Anthropometric Data

Participant's anthropometric data like height (System Dr. Keller I, Längenmesstechnik GmbH, Limbach-Oberfrohna, Germany) and weight (SECA GmbH & Co KG, Hamburg, Germany) were assessed by the study investigator. BMI was calculated by dividing weight in kilograms (kg) by the height squared in meters. Reference values for BMI in children were drawn from the German reference values from Kromeyer-Hauschild et al. (14).

Anamnestic data were obtained from the patients file (original cancer disease and date of diagnosis, treatment regimens, comorbidities). A physician retrospectively computed the amount of administered anthracyclines (mg/m^2^ body surface) and mediastinal irradiation (Gray, gy).

### Health-Related Physical Fitness (HRPF)

The Fitnessgram® is a valid and reliable method to assess HRPF (15). The test battery consists of five motor tasks:

- maximum repetitions in Push-Ups (upper body strength and muscular endurance)
- maximum repetitions in Curl-Ups (abdominal strength and muscular endurance)
- Sit-and-Reach (hamstring flexibility) recorded separately on both sides
- Shoulder Stretch (upper arm and shoulder girdle flexibility) recorded separately on both sides
- trunk extensor strength and flexibility was assessed with the trunk lift (prone position, lifting Shoulders as far from the ground as possible) which was performed twice, but just the better value was recorded.

One valid Push-Up counted when the body was lifted and lowered in a prone position with an elbow flexion ≤90°. Curl-Ups are complete upper body elevations out of a supine position, without the use of the arms (knees are flexed, feet are flat on the floor). Sit-and-reach was assessed in a sitting position, one leg straight and the other one flexed, as distance from fingertips to toes at maximal stretch. For Shoulder Stretch, the participants had to stretch one hand up the back and the other hand from above down the back. The distance between both hands was measured. For statistical analysis the mean value of both sides was calculated. A detailed description of these tasks is available in the online supplement of our recent article (16).

For all of the five tasks, individual z-scores (standard deviation-scores) were calculated using data from the “Sternstunden” cohort of 983 children (498 girls), with a mean age of 11.8 ± 2.3 years. This project was designed to estimate several health outcomes in healthy children in multiple Bavarian schools.

### Hemodynamics and Arterial Stiffness

Blood pressure and arterial stiffness were assessed after 5 min rest in the supine position with a single measurement on the left upper arm using the oscillometric Mobil-O-Graph (I.E.M. GmbH, Stolberg, Germany and HMS Client-Server Version 4.7). Cuff size was adjusted to the upper arm circumference. Based on the recorded brachial pulse, central Systolic Blood Pressure (cSBP) and Pulse Wave Velocity (PWV) were indirectly calculated with the ARCSolver Algorithm.

All raw values were also transformed into z-scores according to established German References. For peripheral systolic and diastolic blood pressure the national German cohort (Kiggs Study) (17) values were used and for PWV, central systolic and diastolic blood pressure the values from Elmenhorst et al. (18) were taken.

### Data Analyses

All descriptive data was expressed as mean values and standard deviation (mean ± SD) or as absolute numbers and percentages if appropriate.

BMI, SBP, DBP, cSBP, PWV were expressed in terms of standard deviation scores (z-scores) according to the references mentioned above. The LMS values and z-scores for the five HRPF motor tasks were smoothed out with a Box-Cox-transformation using R-Studio (version 0.99.879, RStudio Inc.) and the module extensions gamlss (version 3.4-8) and AGD (version 0.34). The total HRPF-score represents the mean of the five motor tasks. Normal distribution of the primary outcome parameter (HRPF z-score) was proven with a Kolmogorov–Smirnov test.

For statistical analysis, a one sample *t*-test with test value “0” was conducted to asses significant differences in z-scores from the appropriate reference. Pearson correlation was used to calculate possible associations between HRPF and arterial stiffness. Unpaired Student's *t*-test were performed to assess differences in the 73 CCS that received anthracycline-containing chemotherapy to those who did not.

Data analysis was performed using IBM SPSS 23.0 (IBM Corporation, Armonk, NY, USA). *P* < 0.05 were considered to be significant.

## Results

CCS showed significant reduced HRPF (z-score: −0.28 ± 1.01, *p* = 0.011) in comparison to healthy peers (Table 1). Most prominent were reductions in flexibility by means of sit-and-reach (z-score: −0.32 ± 1.35, *p* = 0.032), shoulder stretch (z-score: −0.58 ± 1.44, *p* < 0.001), and trunk lift (z-score: −0.45 ± 1.69, *p* = 0.014). 20 (21.7%) of the CCS showed a reduction in HRPF z-scores < 1 standard deviation.

**Table 1:** Study subjects.

	**Childhood cancer survivors (*****n*** **=** **92)**	
Sex (girls)	43 (47%)	
Age (years)	12.5 ± 4.2	
Height (cm)	151.0 ± 21.3	
Weight (kg)	46.3 ± 18.3	
	**CCS (*****n*** **=** **92)**	***p*****-value**
BMI z-score^*^	0.21 ± 1.15	0.102
HRPF (z-score)^****^	−0.28 ± 1.01	**0**.**011**
Curl-ups (z-score)	0.22 ± 1.39	0.152
Push-ups (z-score)	−0.09 ± 0.98	0.415
Sit and Reach (z-score)	−0.32 ± 1.35	**0.032**
Shoulder Stretch (z-score)	−0.58 ± 1.44	** < 0.001**
Trunk Lift (z-score)	−0.45 ± 1.69	**0.014**
Peripheral SBP (z-score)^**^	0.31 ± 1.11	**0.017**
Peripheral DBP (z-score)^**^	−0.30 ± 1.25	**0.040**
Central SBP (z-score)^***^	0.12 ± 1.28	0.408
PWV (z-score)^****^	0.07 ± 1.40	0.649

*BMI, Body-Mass-Index; SBP, systolic blood pressure; DBP, diastolic blood pressure; PWV, Pulse Wave velcovity; HRPF, Health-related Physical Fitness*.

* *Comparison to the German reference from Kromeyer-Hauschild et al. (14)*.

** *Comparison to the German reference from the German KIGGS Study (17)*.

*** *Comparison to the German reference from the Elmenhorst et al. (18)*.

**** *Comparison to unpublished data of 916 healthy German children (see section Patients and Methods)*.

The peripheral SBP of CCS was increased (z-score: 0.31 ± 1.11, *p* = 0.017) and the peripheral DBP decreased (z-score: −0.30 ± 1.25, *p* = 0.040), resulting in an increased blood pressure amplitude (pulse pressure). However, the more direct measures of arterial stiffness from the pulse wave analysis; PWV (*p* = 0.649) and central SBP (*p* = 0.408), were not increased.

Moreover, higher HRPF scores were neither associated with lower PWV (*r* = 0.004, *p* = 0.972) or cSBP (*r* = 0.001, *p* = 0.998), nor beneficially associated with peripheral blood pressure.

There was no significant difference in any of the measured functional parameters when the 73 patients that received anthracycline-containing chemotherapy were compared to those that did not receive cardiotoxic therapy.

## Discussion

This study showed impairments in HRPF in CCS. It also demonstrated an increased pulse pressure as a result of increased pSBP and decreased pDBP in CCS. These findings may result from subtle early changes of the arterial wall stiffness, which cannot yet be detected by the arterial stiffness parameters PWV and cSBP. Both of which were still within the expected range in our study.

Pioneering studies in CCS from the end of the last millennium already outlined deficits in fine and gross motor skills during and after cessation of cancer treatment (19–21). They also claimed that these limitations were linked to the neurotoxic effects of anthracycline (vincristine). However, these limitations were not only limited to motor skills. Physical movement skills like balance, jumping, running as well as muscle strength were also found to be impaired (22, 23). Therefore, the results of deficits in HRPF line up with these previously noted functional and physical impairments (24). The reasons for such impairments are manifold and range from drug neurotoxicity to developmental delay during long periods of hospital admission, sedentary behavior, limited opportunity for active play and overprotection (3, 20, 25). Unfortunately, limitations in motor ability have shown to be a predictor of lower physical activity (25). These low levels of physical activity tend to persist into adulthood such that and CCS are more likely to be inactive and unhealthy adults. As a result their often already increased cardiovascular burden is further reinforced by their unfavorable lifestyle preferences (26). Thus, the establishment of a healthy and active lifestyle is an important treatment goal in order to improve long-term morbidity in these patients (26).

While inactivity is a predictor of cardiovascular morbidity and mortality *per se* (27), reduced physical activity also leads to diminished physical fitness. Both parameters have been shown to be strongly associated with cancer mortality in adults (28) and also improving both, must be part of a successful rehabilitation programs in CCS. It is unlikely that these limitations will normalize without treatment, although Hartman and colleagues reported on normalization in HRPF in CCS 5 years after treatment (4).

- Our finding of an elevation of the pSBP in CCS demonstrates that early signs of chemotherapy-induced cardiovascular damage can already be detected in early childhood. Our results are consistent with recent studies which have demonstrated elevated pSBPs and (pre) hypertension as late complications of cancer treatment regimens (29, 30).
- In addition, the significantly decreased pDBP in combination with the increased pSBP results in a high pulse pressure, which is a known risk factor for CVD and is mentioned in the current guidelines for arterial hypertension (31, 32). In the elderly, every 10 mmHg increase in pulse pressure increases the risk of cardiovascular events by 13–22% (33). Unfortunately, this has not being translated into z-scores. Increased pulse pressure has also been reported in male acute lymphoblastic leukemia survivors by van Waas et al. (29).

Surprisingly, arterial stiffness (as measured by PWV and cSBP), an early, subtle marker for CVD (31), was still within the normal range according to these measurements in our CCS cohort. This is even though a higher risk for early vascular (endothelial damage/ higher arterial stiffness) and biological aging has previously been reported for such patients (9, 10). The major culprits involved in this premature aging are anthracyclines. These chemotherapy drugs have both cytotoxic and cardiotoxic effects (34). Two research groups clearly state the influence of anthracyclines on increased arterial stiffness and impaired endothelial function (9, 10, 35). Another paper reported only a significant influence of radiation, but not chemotherapy, on arterial stiffness parameters (36). Our findings are in line with the results from the SurFF program. This program only found a significant increase in the PWV in CCS older than 18 years as compared to controls. They, like us, did not find a significant increase in this measurement in children. Therefore, the authors suggested an exponential worsening of the PWV with increasing age in CCS (37). However, all these results have to be handled with caution because our current knowledge about the detection of markers for early subclinical cardiovascular damage and their changes over time in CCS is complicated as a result of a number of factors including: heterogeneous study groups, variation in applied methods, use of differing surrogates for measuring arterial stiffness, and varying lifestyle habits between the CCS.

From the hemodynamic point of view, only the increased pulse pressure possibly reveals early vascular aging in our CCS cohort, whereas the limitations in HRPF were manifest. Regular physical activity has a pivotal role to play in the prevention of both of these factors. Not only does it improve the HRPF, it also improves endothelial function and NO synthesis by the creation of shear stress. Several studies underline this theory and the importance of physical activity as part of successful rehabilitation (3, 4). However, this is a multifactorial approach where physicians, physiotherapists, psychologists and family have to work together.

## Conclusion

In accordance with previous studies we found the HRPF to be slightly impaired in CCS. We also found the pulse pressure, but not the PWV, to be elevated in CCS. This combination of reduced HRPF and impaired vascular parameters may raises the likelihood of an increased long-term CVD risk in these patients. This should be taken into consideration when planning tertiary prevention measures for CCS.

## Limitations

The cohort presented in this study differ widely in number of ways including cancer types, treatment regimens and doses used, age at diagnosis, and time since diagnosis. However, the approach used to assess the questions asked seems reasonable when taking into account recent studies/reviews, which also report similar findings in similarly heterogeneous CCS cohorts (38). Patients were tested under non-fasting conditions what might have influence on vascular assessment.

## Data Availability

The datasets for this study will not be made publicly available because Data safety and protection act.

## Ethics Statement

The study protocol (project number 724-16) was approved by the local Ludwig Maximilian University ethical board.

## Author Contributions

PvK was responsible for coordination, examination and collecting data, analyzing and processing of the data and drafted the manuscript. JM was responsible for conception and design of the study, analyzing and processing the data and gave important input for drafting and revising the manuscript. CQ and LT coordinated examinations and collected data. RO and TF gave important input for drafting and revising the manuscript. IS performed medical examinations and gave important input for drafting and revising the manuscript. All authors have read and approved the final version of the manuscript.

### Conflict of Interest Statement

The authors declare that the research was conducted in the absence of any commercial or financial relationships that could be construed as a potential conflict of interest.
